# Learnings in Digital Health Design: Insights From a Pilot Web App for Structured Note-Taking for Patients With Rheumatoid Arthritis

**DOI:** 10.2196/49358

**Published:** 2023-11-28

**Authors:** Ujwal Srivastava, Shobha Dasari, Neha Shah

**Affiliations:** 1 Department of Computer Science Stanford University Stanford, CA United States; 2 Division of Immunology and Rheumatology Stanford School of Medicine Stanford, CA United States

**Keywords:** digital health, biodesign, technology, software, web app, codesign, patient empowerment, note-taking, medical information, web application, web-based, technology engagement

## Abstract

**Background:**

Patients fail to accurately remember 40% to 80% of medical information relayed during doctor appointments, and most standard after-visit summaries fail to effectively help patients comply with behaviors to manage their health conditions. The value of technology to empower and engage patients in their health management has been shown, and here we apply technology to help patients remember and act upon information communicated during their medical appointments.

**Objective:**

We describe the development of WellNote, a digital notebook designed for patients to create a customized plan to manage their condition, plan for their appointments, track important actions (eg, medications and labs), and receive reminders for appointments and labs.

**Methods:**

For this pilot, we chose to focus on rheumatoid arthritis, a chronic condition that relies on many of these features. The development of WellNote followed a structured method based on design thinking and co-design principles, with the app built in close collaboration with patients and a physician partner to ensure clinical relevance. Our design process consisted of 3 rounds: patient and physician interviews, visual prototypes, and a functional pilot app.

**Results:**

Over the course of the design process, WellNote’s features were refined, with the final version being a digital notebook designed for patients with rheumatoid arthritis to manage their health by helping them track medications and labs and plan for appointments. It features several pages, like a dashboard, patient profile, appointment notes, preplanning, medication management, lab tracking, appointment archives, reminders, and a pillbox for medication visualization.

**Conclusions:**

WellNote’s active and structured note-taking features allow patients to clearly document the information from their physician without detracting from the conversation, helping the patient to become more empowered and engaged in their health management. The co-design process empowered these stakeholders to share their needs and participate in the development of a solution that truly solves pain points for these groups. This viewpoint highlights the role of digital health tools and the co-design of new health care innovations to empower patients and support clinicians.

## Introduction

### Background

Patients often struggle to remember key information communicated during their medical appointments, with some studies estimating that 40% to 80% of medical information relayed by physicians is forgotten. Key factors may be diagnosis-related stress and forgetfulness, which limit patients’ retention of peripheral information and adherence to treatment, labs, and follow-up appointments [1,2]. As a result, patients find it difficult to manage their conditions with only a limited understanding of their diagnosis, resulting in failure to adhere to medical recommendations [3].

Previous studies have shown the efficacy of providing patients with access to their clinical visit notes. In a survey of 136,815 patients at health systems who participated in OpenNotes pilots, DesRoches et al [4] concluded that reading clinical notes helped many patients manage their medical conditions, with 14% of patients reporting that reading their notes made them more likely to take their medications as prescribed, and 33% of patients reporting that their notes were extremely important in assisting with their regimens.

Though there are many technological tools addressing the problem of clinician note-taking and documentation (eg, Suki AI, Dragon AI, and Microsoft Nuance), there are few existing tools that enable patients to take notes about their medical visits. Manta Planner and Abridge are 2 well-established patient-facing tools for appointment documentation. Manta Planner serves as a medical planner for patients with cancer to track and manage symptoms, medications, nutrition, and notes for their care team [5]. However, Manta Planner is a physical notebook, which limits its use as a tool for patient-physician communication outside of visits. Abridge automates the clinical documentation process for clinicians and sends an artificial intelligence–generated after-visit summary (AVS) to patients [6]. As a summarization tool, Abridge has limited scope for patient input and engagement at the time of medical decision-making. This may hinder patient adherence to the actions and lifestyle changes delineated in the notes.

To combat these issues, we built WellNote, a web application that acts as a digital patient notebook. The application has 2 main goals: to facilitate information tracking and storage following medical appointments and to aid patients in treatment adherence. Patient education has been shown to improve engagement and outcomes, and note-taking is a key step toward promoting health literacy [7].

Technology-enabled solutions can play a crucial role in addressing these issues. Despite the rise in health care technology, coproduction with physicians, patients, and other relevant stakeholders in health care has lagged behind [8]. Digital health solutions are often built in isolation from the systems and communities they intend to serve [9,10]. Furthermore, some patients are using technology to self-educate about their health, discover the best treatments for their conditions, and optimize their care. The rise of this empowered group of patients, known as e-patients, signifies the desire of patients to be active stakeholders in their care journeys [11]. Going forward, physicians and patients need to be at the core of the design process, ensuring that digital health solutions meet their needs and are clinically relevant.

The aim of this paper is to describe the design process of a web app to facilitate structured note-taking for patients with rheumatoid arthritis (RA). RA was selected for a proof-of-concept product because it is a chronic condition that requires patients to be highly involved in managing their care. This involves tracking concrete and longitudinal information, such as labs, regular medications, and pain symptoms (ie, morning stiffness), that can be well-documented in a digital notebook. The majority of chronic condition management happens outside of a health care facility, and patient literacy and understanding of their condition is especially important in these contexts.

In this viewpoint, we provide a visual display and demonstration of the proposed workflow, cover the rationale for key features of the app, reflect on our application of design thinking principles to create a user-centered product, and connect the lessons of our app to broader themes in digital health.

### Overview of the Technical Solution

WellNote is a digital notebook for patients with RA to manage their health condition. To help patients remember the information conveyed by physicians during appointments, our web app aims to help patients track important actions, such as medications and labs. The application also aids patients in planning ahead for their appointment through the preplanning feature, which allows patients to write down any health developments or questions they wish to discuss with their provider before their appointment.

WellNote has an intuitive design with the following pages and functionalities (Multimedia Appendix 1):

1. Dashboard: This page contains links to the patient’s profile, upcoming appointments, and a graph of recent pain scores self-reported by the patient.
2. Profile: This page contains the patient’s name, contact information, diagnosis, insurance information, preferred lab, and preferred pharmacy.
3. Appointment: This is the core note-taking pipeline that patients use for each clinic visit and includes the following 3 components:
  Preplan: The preplan asks patients about recent symptoms, medications and side effects, morning stiffness, pain score, and questions for the physician. This tab is intended to be filled out before the appointment.
  Medications: This component allows patients to add, edit, or delete medications using a prepopulated drop-down menu.
  Labs: This component allows patients to add, edit, or delete labs using a prepopulated drop-down menu. This is integrated with Google Calendar to remind patients about lab appointments.
4. Past appointments: This is an archive of previous appointments, each containing the preplan, medications, and labs associated with that appointment. Patients can access the information from these appointments at any time.
5. Reminders: This page contains upcoming labs and medication reminders (eg, refills).
6. Pillbox: This is a visually intuitive display of a patient’s medications for each day of the week, including dosage and schedule.

**Figure 1:**
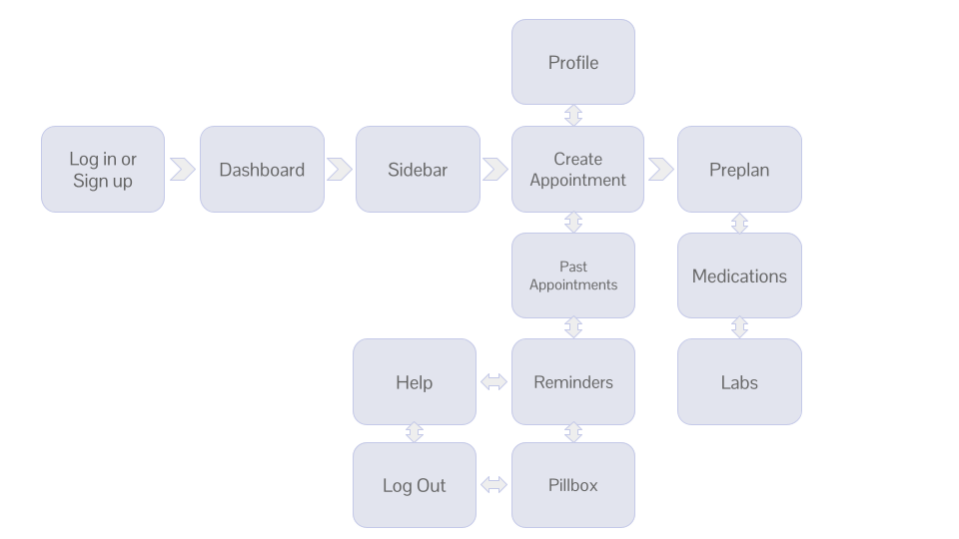
WellNote user flow schematic.

In a typical workflow, after logging in, users will arrive at the dashboard where they have the option to navigate between several pages using the sidebar. Before an upcoming visit, patients can create a new appointment and fill out the preplan. During the appointment, they can take notes on medications and labs using the structured format. At any point in time, patients can view upcoming labs, their past appointments, their current medications, and update their profile (Figure 1).

## Methods

### Co-Design With Physicians

Our web app was built in close collaboration with a physician partner so that our notebook’s workflow could complement the typical course of visits in their clinic.

#### Round 1: Interviews

We initially approached our physician partner with a conceptualization of the problem domain from the patients’ perspective. Through our conversations with our physician partner, we were able to refine the scope of the problem, identify the highest-leverage opportunities to address the problem, and co-design a product that was tailored to their existing clinical workflows.

Given the limited time physicians have with their patients, streamlining visits was a top priority elicited during interviews. To achieve this, we received a suggestion to add a preplan feature to the appointment workflow to encourage patients to engage with their symptoms, medications, concerns, and questions before coming to the doctor’s visit to use clinic time more effectively. For RA applications, our preplan asks about pain scores on a scale of 1 to 10, a common question used by physicians. Pain scores were used to generate a graph on the dashboard to give a quick and visually intuitive review of recent check-ins. To further streamline patient note-taking, our physician partner suggested using drop-down input options, as opposed to free-form, unstructured text entry.

#### Round 2: Visual Prototype

Based on this feedback, we created a visual prototype of the proposed solution using presentation slides and graphics to receive specific and tactical feedback.

Our first prototype had one continuous open-ended section for note-taking, but our physician partner suggested separating tabs for medications and labs, the primary patient actions discussed during RA visits. The physician’s concern about the lack of adherence to labs led to the development of a Google Calendar integration so that patients could receive automatic calendar reminders to schedule and attend their lab draws, a feature that is missing from other clinical documentation tools, such as EPIC [12].

Through co-designing with a physician, we discovered design needs from a clinician perspective that we would not have known otherwise. This improves our app’s ability to integrate into clinical practice smoothly and fill physician-identified holes in care.

#### Round 3: Functional Pilot App

We presented our finished pilot app to our physician partner, who reviewed it positively. They said the design was user-friendly and the product would easily integrate into their clinical practice. Further product requests included note-taking tabs for lifestyle recommendations, including diet, exercise, and sleep, using drop-down menus with several common recommendations for RA patients, though we saved this feature for a future round of development.

### Co-Design With Patients

As we received insights from patients, the goals and scope of the product shifted.

#### Round 1: Interviews

Initially, we envisioned a tool to facilitate patient notes, and our first idea was a free-text note-taking tool with smart annotation features to be used during appointments, which would hyperlink based on key terms to verified resources on symptoms and medications. However, through patient interviews we discovered that patients find note-taking to be distracting during appointments and that a clinician-generated AVS is intended to address similar tasks, though with varying levels of success.

Upon receiving this feedback, we revised the solution to be a tool that uses natural language processing or ontologies to automatically provide annotations and action items after a patient uploads their AVS. However, this solution was hindered by inconsistent patient access to a clinician-generated AVS. In addition, given the 10-week timeline for development and testing, we decided against training a language model to generate this information until a future round of development.

#### Round 2: Visual Prototypes

Ultimately, the team decided to pursue building a patient notebook with structured drop-down menus for inputs to minimize the note-taking burden on patients while allowing for the effective documentation of information from the appointment. Patients believed that the process of documenting their medications, labs, and questions at the end of the appointment would help them understand what was discussed with their physician and remember their required actions after the appointment.

Table 1 shows samples of feedback received from patients about the visual prototype in the product development process. The involvement of patients in our design process allowed us to refine the problem definition based on what patients prioritized most. From there, we adapted our solution idea to more appropriately address this new framing of the problem.

**Table 1:** Patient feedback on WellNote app visual prototypes.

Name	Feedback	Implemented changes
Patient 1	Would appreciate the ability to take notes, but maybe not during the appointment–logging talking points before the appointment and action items at the end. Digital pillbox looks really helpful–not sure how this differentiates from other tools but would be helpful. Her pain points around remembering to call her pharmacy to refill her medication, go to get her labs done, call her doctor to book an appointment–would appreciate automation to just do the things for her (or at least having the phone numbers loaded into the app to avoid searching up every time).	Added the ability for patients to take notes before the appointment Added a structured field for action items at the end of the appointment Added the ability to edit notes after the appointment
Patient 2	Likes the idea of taking notes–web app is not the best format, but mobile would work. Wants integrations with email or text for the notifications because that’s where she checks most–phone notifications are great too. HIPAA the Hippo is cute :) If medication and exercise are added manually, are the calendar notifications able to be customized with descriptions, frequency, etc.?	Added Google Calendar integration for appointments and labs to show up

#### Round 3: Functional Pilot App

We demonstrated the working prototype to our patient partners, who navigated the app interface and gave us feedback on whether we had addressed their needs and the issues discussed in earlier rounds of co-development. Table 2 provides a summary of the feedback we received in this successive round.

**Table 2:** Patient feedback on the WellNote functional pilot app.

Name	Feedback	Implemented changes
Patient 1	Pain score tracking is a nice feature–wants the ability to track it between appointments too. That information would be helpful for their doctor to see as well to know how they’re doing between appointments. Wants the doctor to be able to directly access the preplan information in their medical record system. Mobile app format would be great for this. Phone notifications will help with remembering to take the medications.	Added a pain display on the dashboard to see the trajectory of pain between appointments
Patient 2	Wants this to be mobile-responsive or a mobile app–getting phone notifications will be really useful for her. She also brings her phone to all appointments, wouldn’t necessarily bring her laptop. Pillbox will help with remembering to take multiple medications. Being able to read over past appointments is helpful to see their disease progression and remember previous visits. Getting reminders to fill out the preplan a few days before the appointment would be helpful.	Added upcoming appointments and reminders views on the dashboard Added a mobile application into the product roadmap

### Ethical Considerations

We followed the Stanford School of Medicine institutional review board protocol and determined that this feasibility pilot did not meet the criteria for research under 45 CFR 46 and therefore, did not require institutional review board review or informed consent. The feedback about the app that was collected contained no personal health information and was deidentified, and its explicit purpose was for quality improvement of the app.

## Results

### Overview of Results

**Figure 2:**
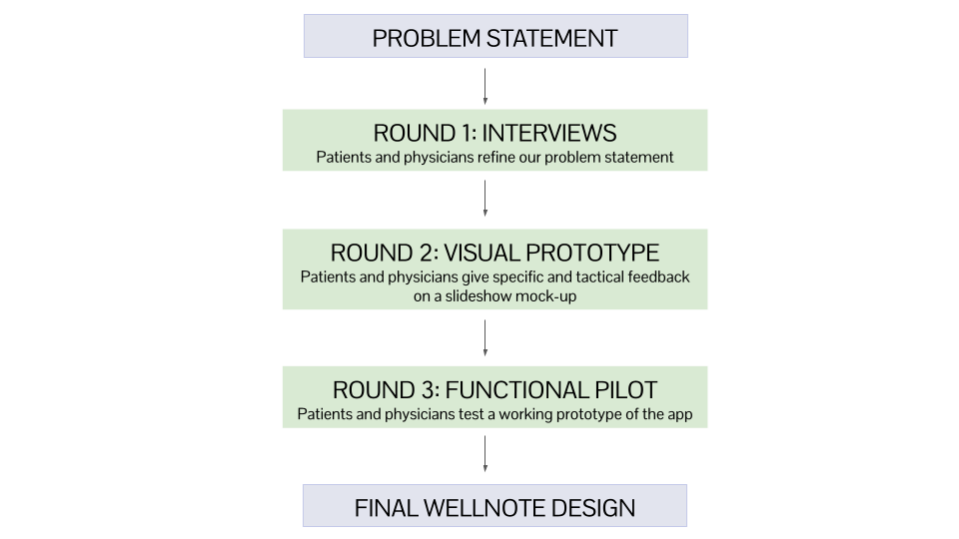
WellNote development process.

We followed the development and testing process outlined in Figure 2. It is important to highlight that the process involved 3 stages: interviews with key stakeholders, visual prototyping, and functional pilot app testing. At each step, patient and physician input was crucial to refining our project and arriving at the final working app.

The app has the following pages: dashboard, profile, preplan, medications, labs, past appointments, reminders, and digital pillbox. Together, these pages help patients before, during, and after appointments by facilitating mindful planning ahead, easy-to-follow note-taking, and follow-up reminders for key action items.

WellNote was created to integrate into a clinic workflow and meet the needs of physicians and patients by maximizing usability and minimizing how time-consuming it is. Accordingly, several key design decisions reflect this intention. First, note-taking was designed to be as structured as possible, making use of drop-down menus and short-answer questions when possible. For instance, the labs and medications tabs were populated by relevant drop-down options (as opposed to free-answer boxes) to make it easier and faster for patients to track critical information, like medicine names, lab types, and related instructions. This format minimized screen time and distractions to patient-physician communication while facilitating note-taking and understanding of complex jargon and directions. To help patients follow up and adhere to recommendations from doctors, we implemented a Google Calendar integration feature to provide patients with customized reminders for labs, such as fasting instructions, lab appointments, and lab locations based on previously indicated preferences. The pillbox automatically updated whenever changes were made to medications in the appointment workflow to accurately reflect the patient’s current regime.

To narrow our scope, we decided to focus on building a digital notebook for a specific use case, namely, patients with RA. The most common medications, labs, and physician examination questions for RA were used to prepopulate the appointment workflow. While the current version is RA-specific, WellNote can be easily modified for other conditions, as its adaptable and modular design makes it transferable to other physicians’ clinical practices and preferences.

### Data Security and Privacy

Since WellNote is an early-stage proof of concept, there were no collected data at the time of presenting this viewpoint. However, data security and privacy are paramount, and here we include an overview of hypothetical use cases. Since the app would be deployed in a clinical setting, the data would be stored securely in the hospital’s or clinic’s database, which is encrypted and Health Insurance Portability and Accountability Act compliant. Each piece of information requested was relevant to the physician and was validated by our physician partner in this version of WellNote. Additionally, all entry fields in WellNote are optional to complete, so patients can choose what they would like to share with their physician and what they would like to opt out of sharing. 

## Discussion

### Summary of Findings

WellNote’s core functionality is a notebook to improve patient engagement that gives patients tools to track and streamline their appointments, plan for their appointments in advance, and log their medications and upcoming labs. By putting all the information in one organized place and generating reminders for upcoming deadlines, our app aims to address issues of information retention, treatment adherence, and the lack of patient-facing products in this domain. Through the development of this patient tool, our team followed a structured method for product design based on design thinking principles [13,14]. The insights gained from our physician and patient interviews were reflected in conscious design choices, and this process serves as an example of digital health development in partnership with health care stakeholders.

### Interpretations, Implications, and Comparison to Existing Literature

Many standard AVSs are not patient-friendly, digestible, or actionable. Often, these documents are poorly structured, full of medical jargon, and not aesthetically appealing. The information in these documents is hard to parse, and clinicians struggle to customize the electronic health record–provided AVS to their patients’ preferences [15]. WellNote’s design helps remedy this.

One design consideration was the benefit of active note-taking versus clinician-generated AVS annotation. Patients, medical students, and a physician highlighted some drawbacks of real-time, free text note-taking. This process can be time-consuming in an already truncated clinic appointment, may disrupt the patient-physician conversation by introducing a digital screen, and may duplicate information present in the existing AVS.

At the same time, we received feedback from patients and a physician in support of the structured note-taking model. The active engagement from patients during the note-taking process allows for better understanding and retention, which is missing from summarization-only approaches. Functionality focused on reminders and notifications was prioritized by patients. Our adapted solution addresses many of these points and claims further advantages.

### Addressing Patients’ Needs Around Reminders for Patient Engagement

Our app is not focused on full-fledged note-taking, but rather hones in on facilitating structured logging of action items. Specifically, our design pivoted to focus on preappointment and postappointment support and creating reminders for action items and engagement.

Structured drop-down menus with autopopulated medications, labs, days of the week, and other information reduce the burden on patients. Free text is minimized, making the design user-friendly. Structured note-taking has also been shown to reduce medical errors [16].

### Active Engagement From Patients in the Information Creation Process

Note-taking during the time of the appointment allows for maximum patient engagement. Here, we consider a series of hypothetical scenarios.

#### Hypothetical Scenario 1

Two days before an appointment, the patient is notified to use the preplan feature to document information about their condition since their last visit. In the preplan flow, the patient notes feeling more pain than usual and documents the start of a new medication. During the appointment, the physician and patient are able to explore if there is a correlation between the new medication and increased pain and create a plan of action together. This example illustrates how the preplan feature can be used to maximize patient-physician time together and empower patients to play a role in decision-making.

#### Hypothetical Scenario 2

During an appointment, a physician increases the dose of a medication by 20 mg. Whereas previously this adjustment might have been mentioned without much discussion and written in the AVS, the act of note-taking can enable the patient to pause, reflect, and initiate a conversation about their medication regime (eg, they feel certain side effects due to this medication). One of the main advantages of real-time information tracking is that it prevents patients from passively accepting changes, instead equipping them to be e-patients.

### Limitations of the Summarization Approach

Approaches focused on the annotation of existing clinical notes, such as the AVS, are useful and merit consideration. However, many patients currently do not have access to their clinical notes [17]. While the OpenNotes movement has gained traction and policy efforts to increase medical note access are underway, apps focused on summarization exclusively are not reliable. In addition, these solutions fundamentally miss an opportunity to engage patients in the information creation process. Rather than treating them as entities that need a summarized version of their care plan, apps that allow for note-taking can have significant benefits on patients’ perceptions of owning their health and feeling connected and heard.

### Benefits of a Co-Design Approach

By co-designing our digital health tool with patients and clinicians, we were able to build an effective tool that solves a true need for our users [18]. In addition, through patient and physician input in the design of our app, our co-design process encouraged these stakeholders to participate in the development of a solution that they would be willing to use. In the design process, we also formed several key takeaways regarding effective practices when co-designing with clinicians and patients.

This article delineates a process by which to collect patient and physician feedback for an application through interviews, a visual prototype, and a functional pilot application. Through each of these phases, we were able to learn new information about patient and physician needs. From interviews, we were able to receive more generalizable and broad commentary about the most pressing problems facing patients. The visual prototype was a lean mechanism by which we could receive more implementation-specific feedback without building the entire tool. From the functional pilot app, we were able to learn about specific details about additional features that would better help patients. As a result, it was important to design a solution to align incentives and solve related obstacles for multiple stakeholders–WellNote is more useful to patients when clinicians see its value and it fits into their appointment workflow.

Once we had identified and refined our problem space, we found it effective to show an initial prototype for a solution during conversations with patients and a physician. This allowed for more concrete and specific feedback on the prototype than we would have received from a conversation based on ideas alone.

### Limitations

In its current form, WellNote does have some limitations. The choice to minimize optionality in patient note-taking means that the unique circumstances of patients cannot always be captured in drop-down menus. For example, while medication names can be entered using a free-answer box, entering a weekly medication schedule assumes that patients will have the same schedule each week. Allowing the patient to pick more custom schedules can cater to more circumstances, but increases the work and time spent for the patient under the typical use case. Additionally, since patients are expected to enter health information into the app themselves, there can be concerns with information reliability and accuracy. Ultimately, patients should be trusted to be reporters of their medical condition, but patients and physicians should be aware of the potential for error due to mislabeling or confusion. Lastly, our third round of patient feedback (on the functional pilot app) showed that patients preferred a mobile app, so our next round of development will focus on making that transition.

One limitation of our design process was that we only received feedback from 2 patients and collaborated with a single physician, customizing the product to their desired workflow. Though this may limit the generalizability of WellNote to other patients and physicians, the process we followed can be scaled for volume through interviews with more patients and physicians. By interviewing and co-designing our solution with these groups, we were able to design an effective product that solves the needs of multiple stakeholders. This translatable process can be used in the development of other products to solve patient and clinician needs.

### Conclusion

In this viewpoint, we shared details about WellNote, a web app for patients with RA that serves as a digital notebook. By mirroring the flow of an actual clinic appointment, WellNote helps patients plan ahead, track medications and labs after the appointment, and feel empowered and engaged in the management of their condition.

Clinical care is undergoing a paradigm shift where patients are empowered to be actively engaged in their health care. Technology undoubtedly can play a significant role in facilitating patient education and engagement with their care. Perhaps more importantly, the process of designing these solutions must involve patient and physician partners in order to ensure their clinical relevance. The value of tech-enabled patient empowerment has been well-established; going forward, it is time to broaden the discussion to patient-centered tech development.

## Appendix

Multimedia Appendix 1 Images from the WellNote app.

## Abbreviations

AVS: after-visit summary
RA: rheumatoid arthritis

## Data Availability

Data sharing is not applicable to this article as no data sets were generated or analyzed during this study.
