# Supplementary figures and images for: Learnings in Digital Health Design: Insights From a Pilot Web App for Structured Note-Taking for Patients With Rheumatoid Arthritis
Source: JMIR Form Res. 2023 Nov 28;7:e49358. doi: 10.2196/49358 (PMC10716762; doi:10.2196/49358)

Multimedia Appendix 1. Images from the WellNote app.


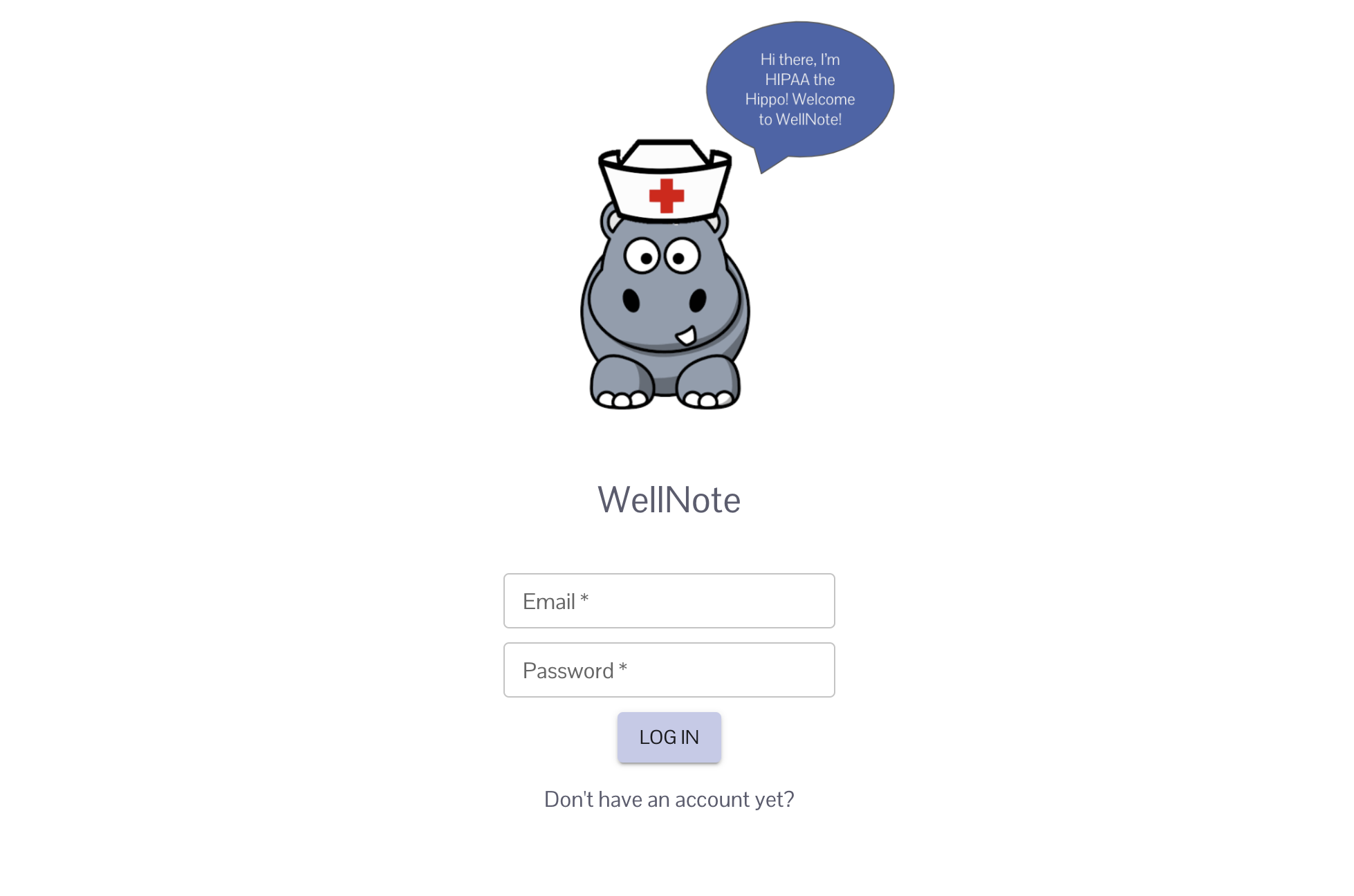

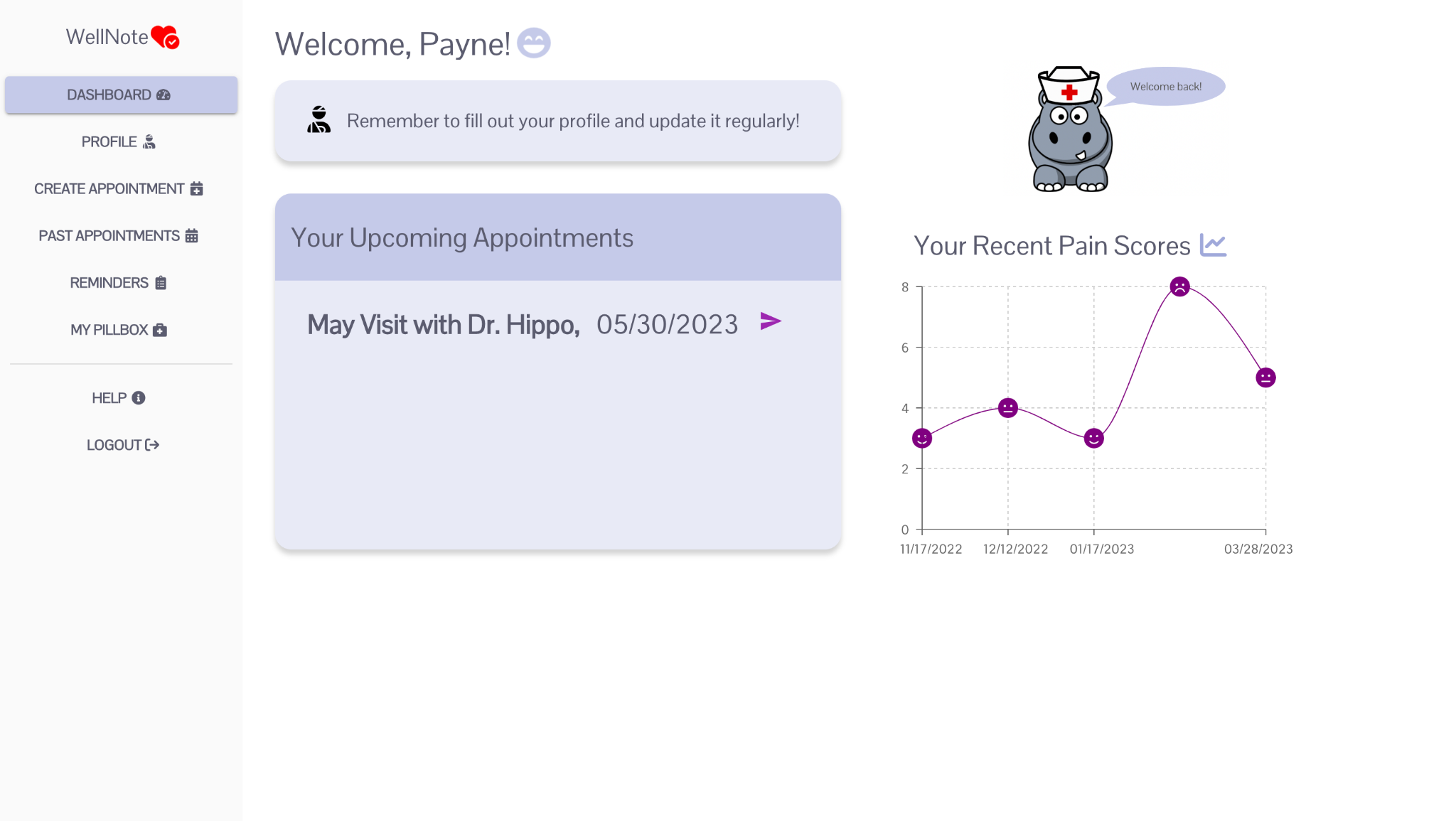


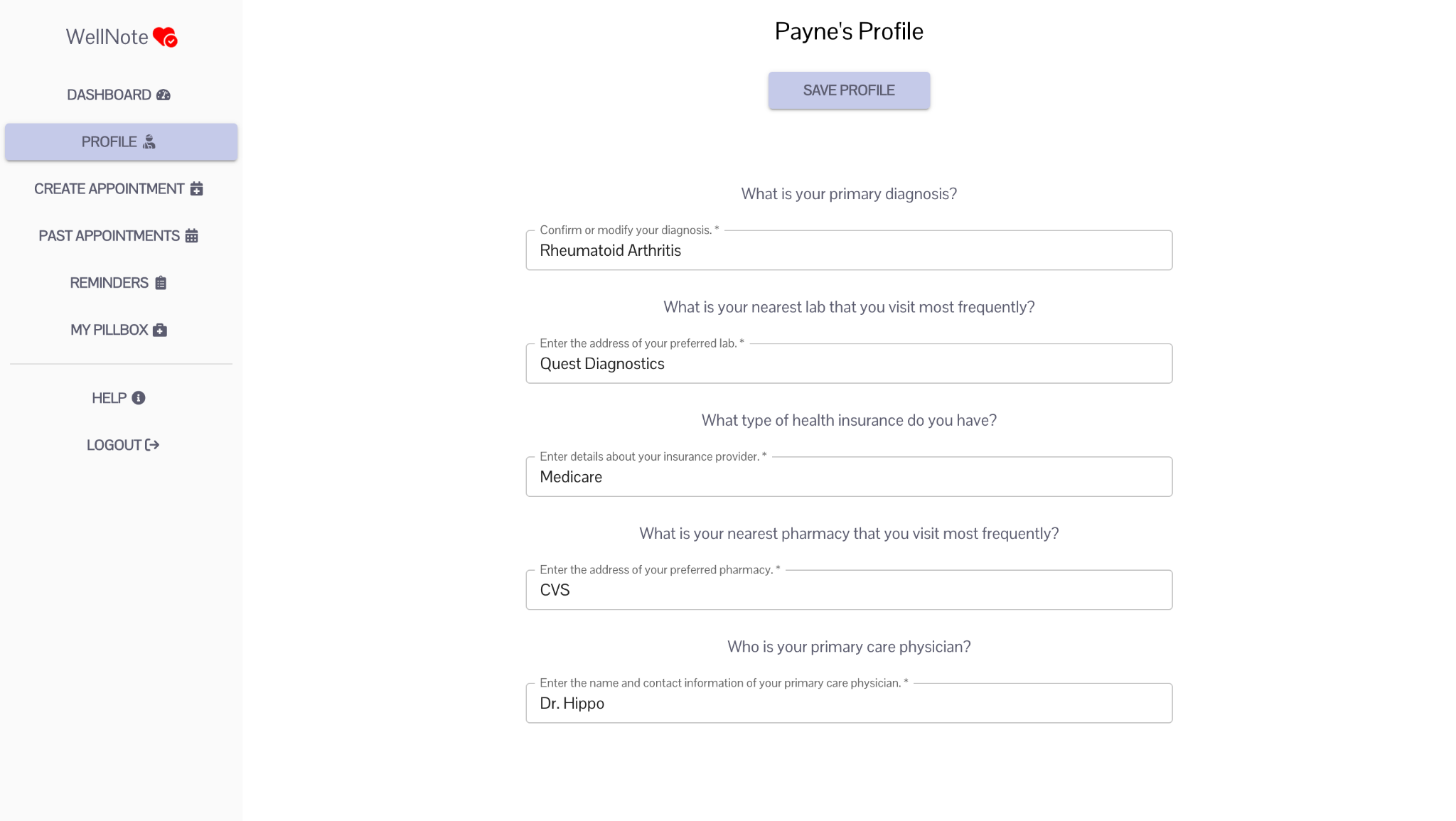

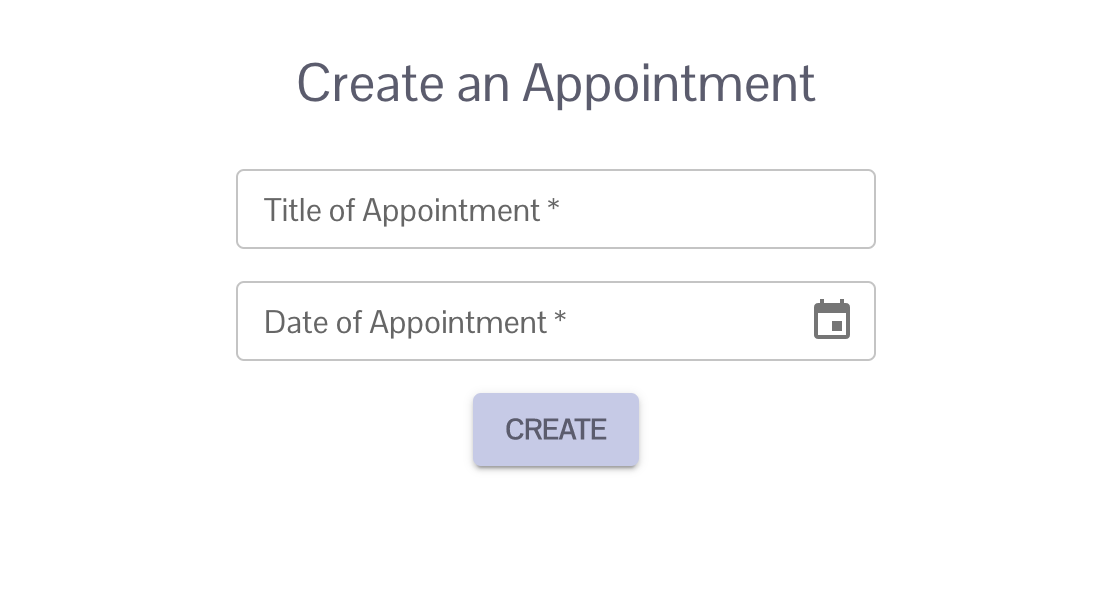


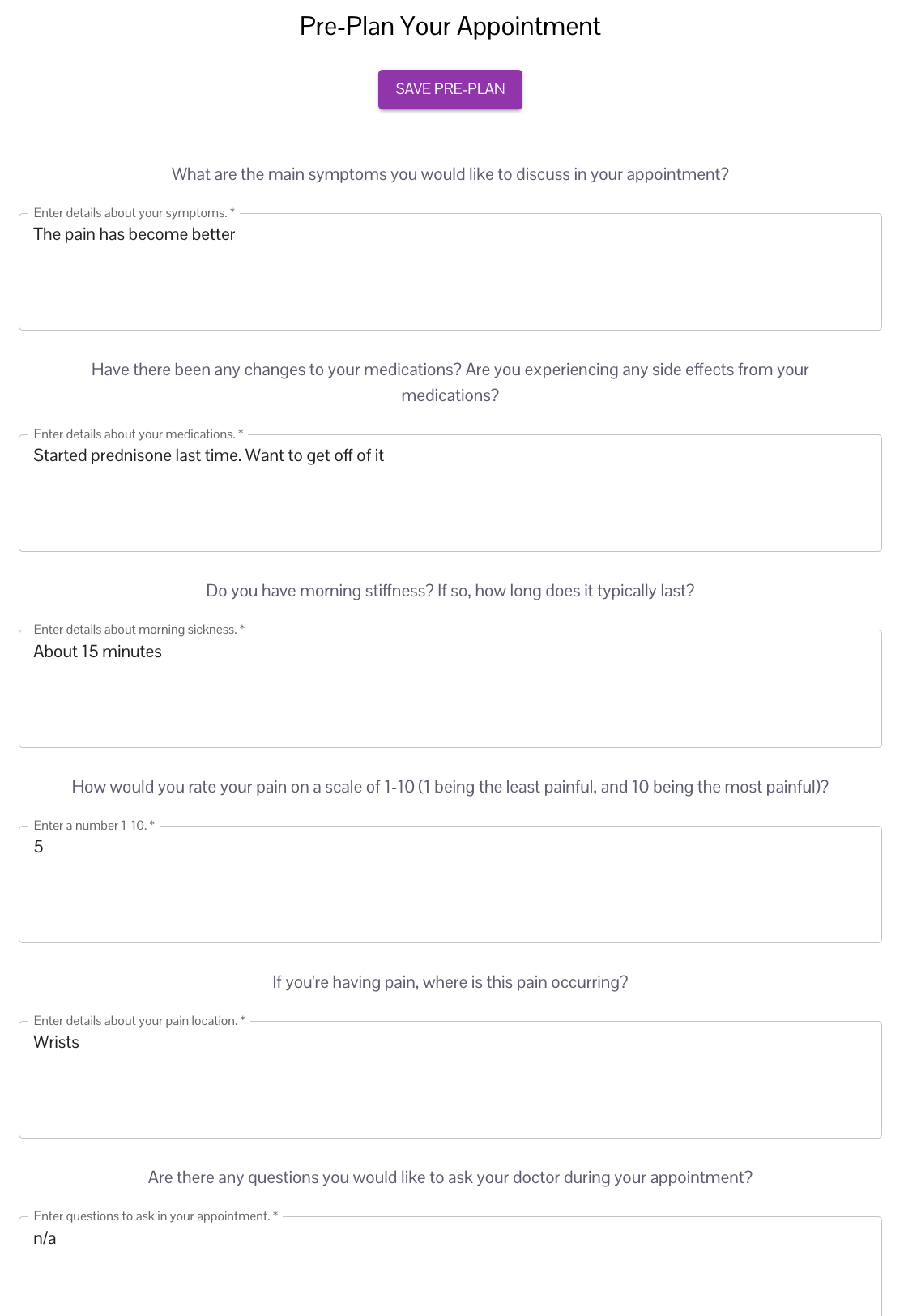

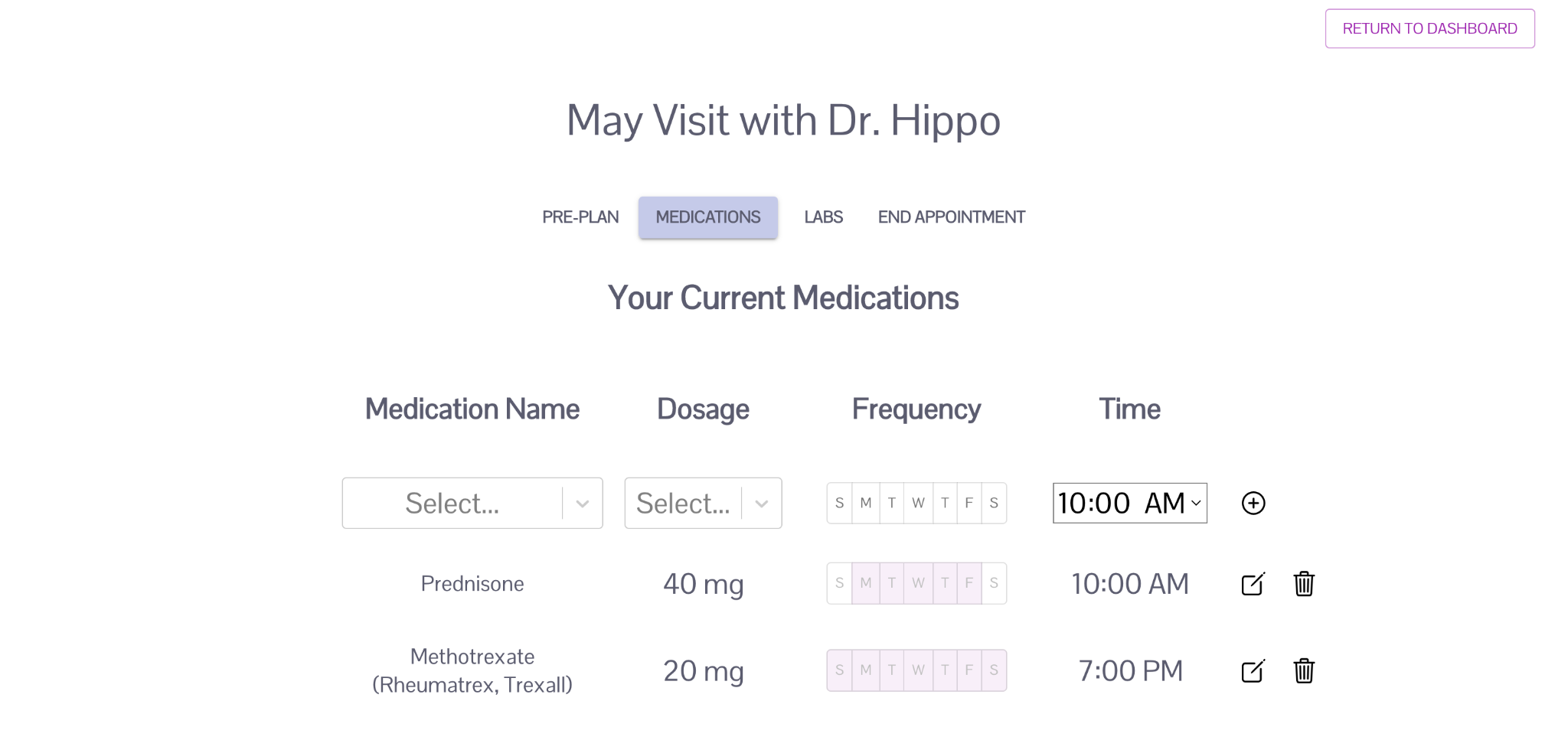

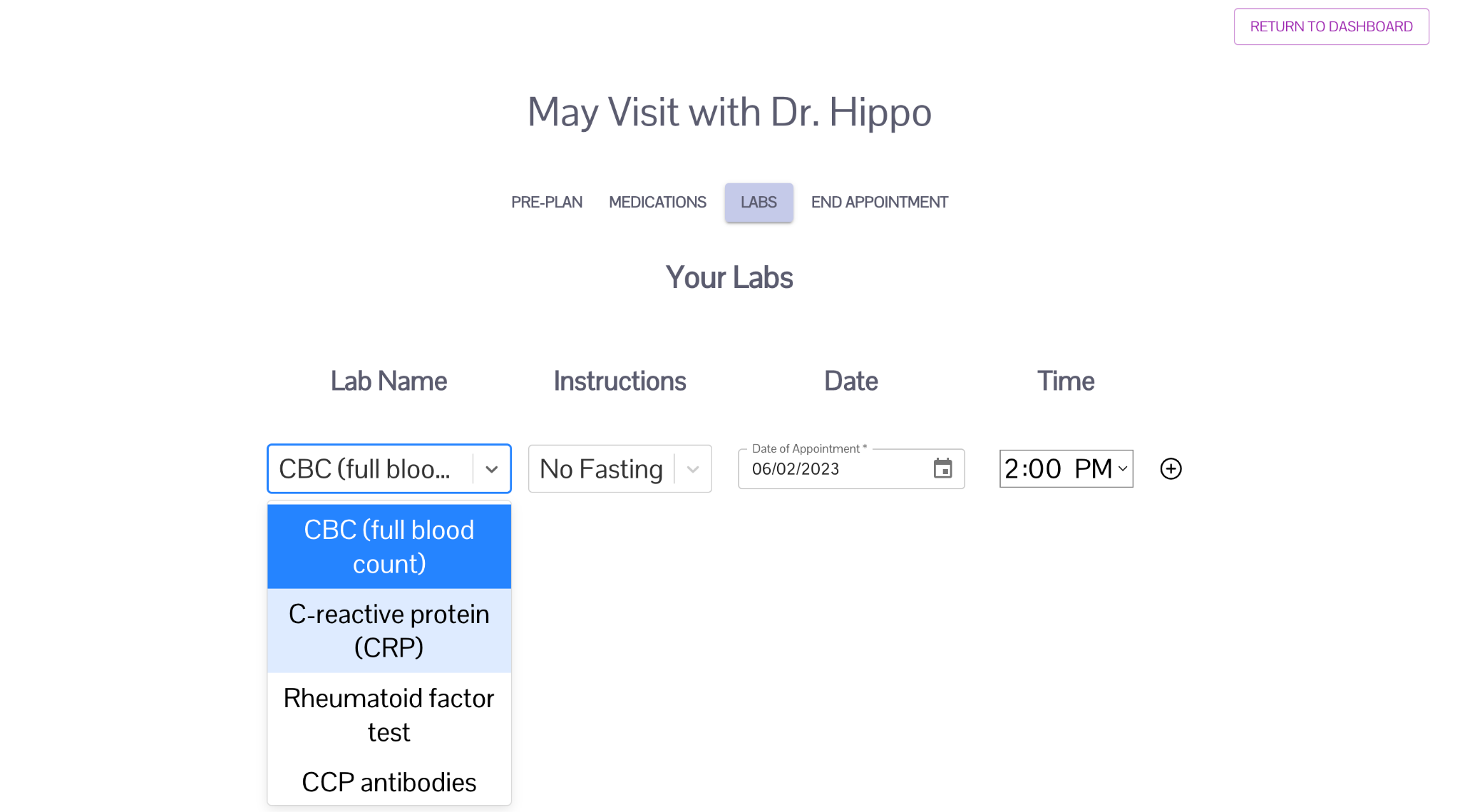

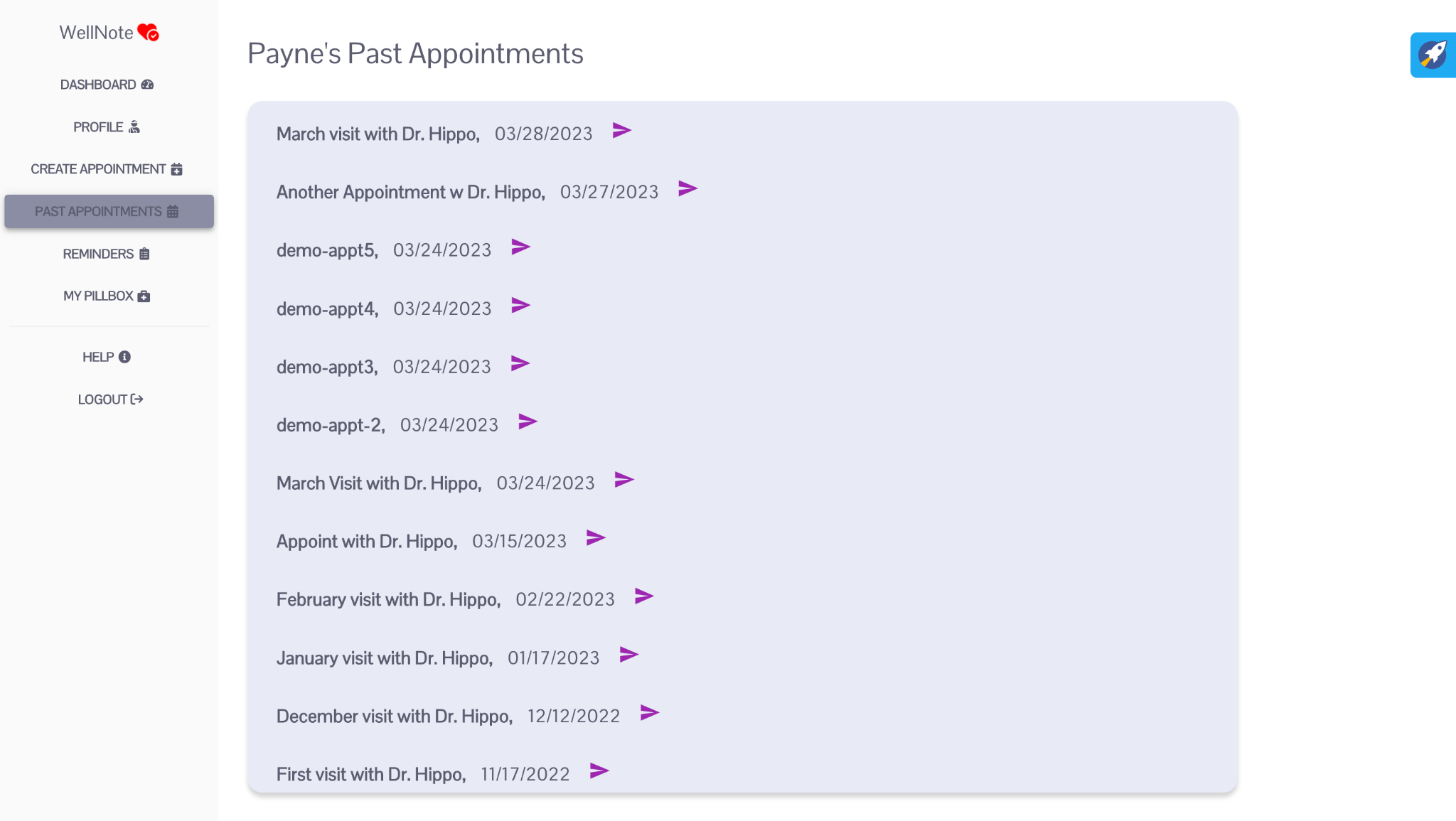


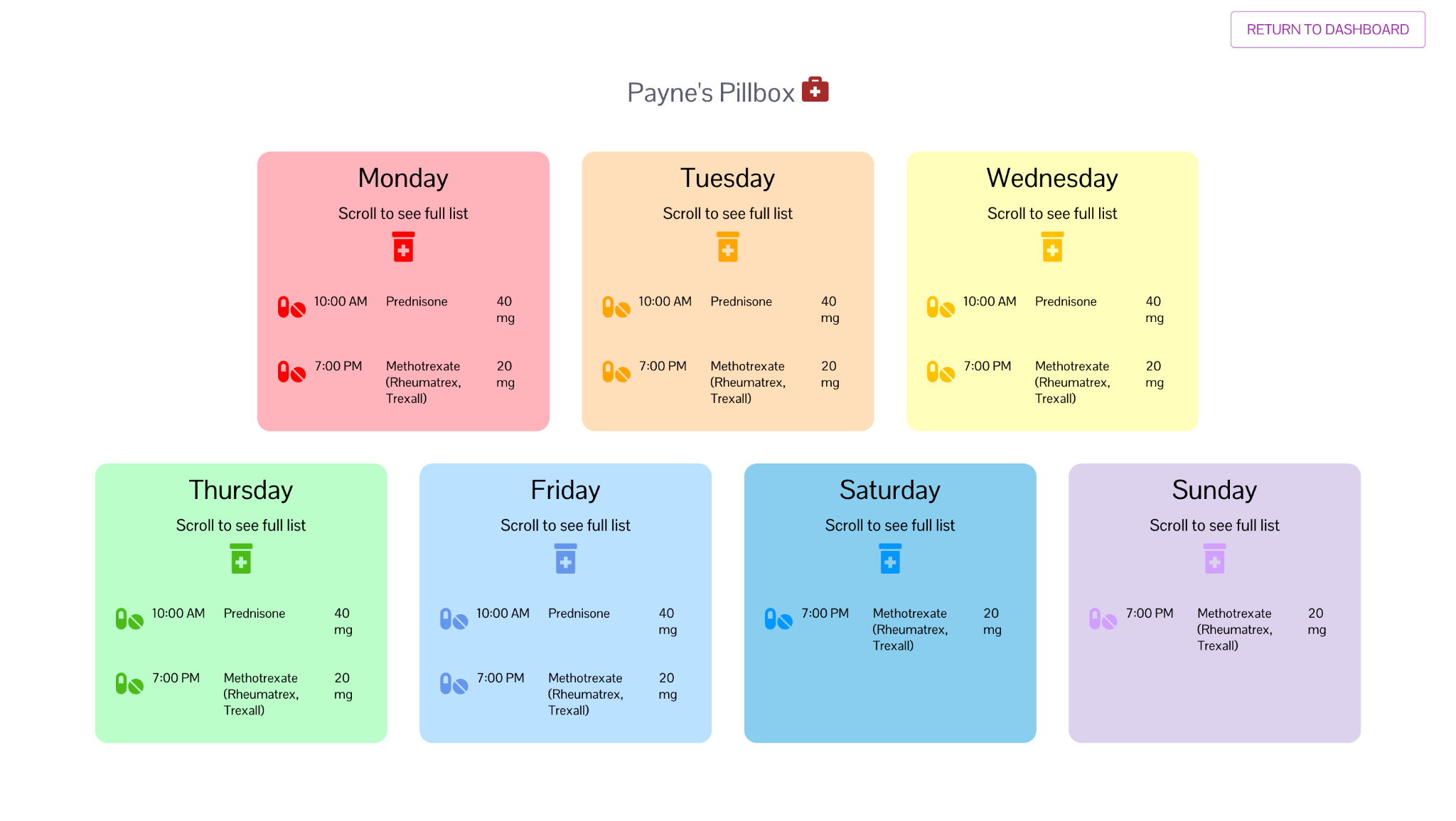

Supplement: Multimedia Appendix 1 [file formative_v7i1e49358_app1.docx]
